# When time matters: a qualitative study on hospital staff’s strategies for meeting the target times in cancer patient pathways

**DOI:** 10.1186/s12913-021-06224-7

**Published:** 2021-03-09

**Authors:** Line Melby, Erna Håland

**Affiliations:** 1 Department of Health Research, SINTEF, P.O. Box 4760, Torgarden, 7465 Trondheim, Norway; 2 Norwegian University of Science and Technology, 7491 Trondheim, Norway

**Keywords:** Cancer, Cancer patient pathways, Hospital, Healthcare workers, Time, Target times, Qualitative methods

## Abstract

**Background:**

Cancer patient pathways (CPPs) were introduced in Norway in 2015. CPPs are time-bound standardised care pathways that describe the organisation of and responsibilities for diagnostics and treatment, as well as communication with the patient and next of kin. The aim is to ensure that cancer patients experience a well-organised, coherent and predictable pathway without any delays in assessment and diagnostics caused by non-medical reasons. Preventing delays in diagnostics by meeting specific target times is central to the successful implementation of CPPs. The aim of this paper is to describe how hospital staff cope with the increased focus on meeting CPP target times and the measures and strategies implemented by hospitals and their staff.

**Methods:**

Data for this paper were collected in a larger study on implementation and experiences with CPPs among hospital staff, general practitioners, and patients in Norway (2017–2020). The study had a qualitative cross-sectional design, and data were collected through interviews. This article is based on semi-structured interviews with hospital staff (*N* = 60) in five hospitals.

**Results:**

Hospital staff are highly aware of the target times, and try to comply with them, in the interest of both the patients and the hospitals. The implementation of CPPs was not accompanied by the allocation of additional resources; therefore, hospitals could not simply increase capacity to meet the target times. Instead, they had to develop other strategies. Four categories of strategies were identified: (i) introducing new roles and more staff, (ii) reorganising the workflow, (iii) gaming the system and (iv) outsourcing services.

**Conclusions:**

Hospital staff are torn between meeting the target times and a lack of resources and capacity. This is not unusual in the current healthcare context, where staff face organisational reforms and increasing demands on a regular basis. It is important to recognise frontline workers’ efforts towards realising new organisational changes. Therefore, carefully weighing the benefits against the costs and undertaking the necessary planning are important in the design and implementation of future care and treatment pathways for patients.

## Background

Cancer patient pathways (CPPs) are time-bound standardised patient pathways that describe the organisation of and responsibilities for diagnostics and treatment, as well as communication with the patient and next of kin. CPPs were introduced in Norway in 2015, and they are based on the national guidelines for cancer diagnostics and treatment [1]. These pathways cover the period from suspicion of cancer to start of treatment. Norwegian health authorities stipulate that the aim of CPPs is to ensure that cancer patients experience a well-organised, coherent and predictable pathway, in the absence of delays in assessment, diagnostics, treatment and rehabilitation that result from non-medical causes [2]. In the Norwegian context, avoiding delays in diagnostics has been highlighted as being particularly important.

CPPs need to be understood within the broader context of organisational reforms in Western healthcare organisations, which have seen increasing standardisation of care delivery [3, 4]. Standardised patient pathways, which are also known as care pathways, critical pathways and integrated pathways, signify the realisation of standard care delivery. Such pathways, of which CPPs are an example, are primarily implemented to achieve more efficient care, to strengthen the quality of care and to reduce variations in cancer care. Additionally, they are also introduced to improve patient safety and patient satisfaction [5–11]. A central feature of standardised pathways is that they streamline processes throughout the patients’ trajectory. This is done by describing activities and defining ideal sub-processes and target times. In this way, pathways can become tools for reducing unwanted variation in and enhancing the quality of care [12, 13].

**Fig. 1:**
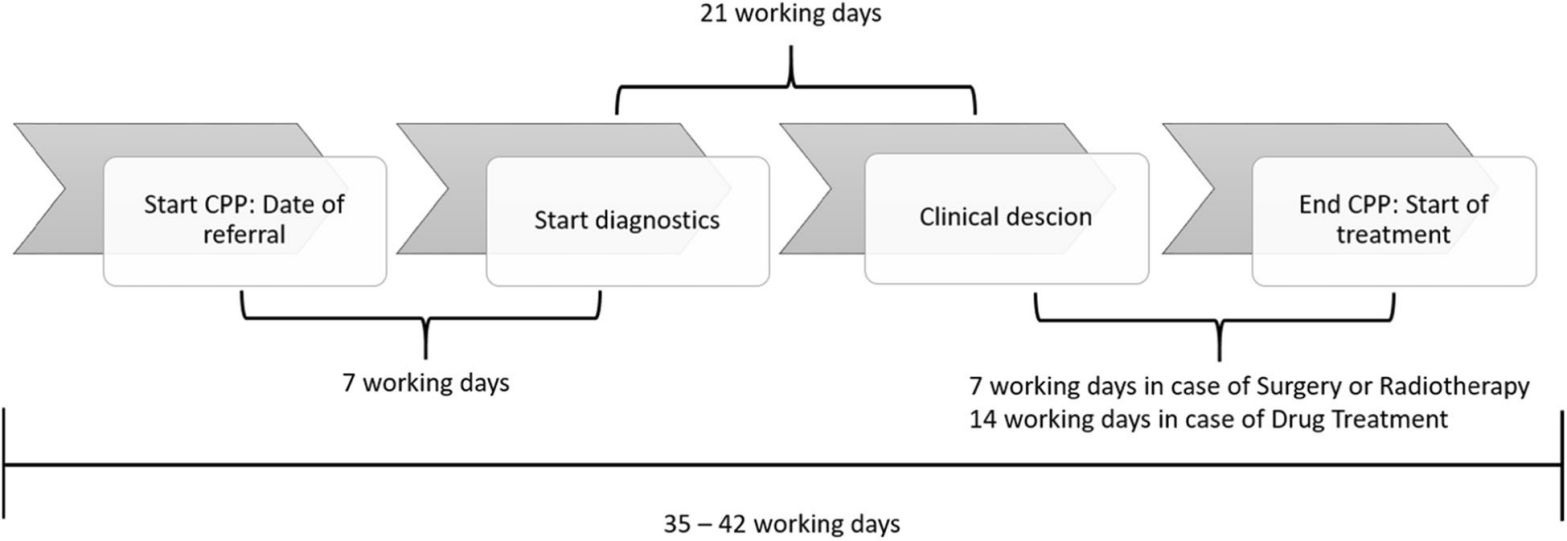
Overview of the CPP for lung cancer

The Norwegian CPPs, like other standardised pathways, are organised in phases. All the CPPs are organised in four phases, and each phase should be completed within a predefined time period, based on the type of cancer. A CPP begins when the hospital receives a referral for a CPP based on a well-founded suspicion of cancer, typically from the patient’s general practitioner, and it ends when the initial treatment starts. The times for each phase are summarised when the CPP ends, in order to determine the total CPP time. The goal is to ensure compliance with the overall target time in the pathway in 70% of the patients. Figure 1 shows a CPP for lung cancer with the target times. From the time the referral is sent, the hospital has seven workdays to start the diagnostics, which includes arranging a number of examinations and communicating with and preparing the patient for them. The diagnostic process should take no more than 21 workdays, and a clinical decision must be made at this point. This means that within 21 days, the hospital needs to determine whether the patient has cancer or not, and in case of cancer, the hospital needs to establish the specific type of cancer and the most suitable treatment. After a clinical decision has been made, there are various times to comply with, based on treatment choice. For lung cancer, surgical treatment or radiotherapy need to be initiated within seven workdays. In the case of patients scheduled for drug treatment (chemotherapy), treatment needs to be started within 21 days.

The target times are normative and not legally binding. However, statistics showing how all Norwegian hospitals comply with the target times for each of the CPPs are published monthly and are publicly available [14]. This attracts the attention of the media, health authorities and hospitals. Furthermore, the anticipated effects of CPPs for the patients are highly dependent on hospitals managing to avoid unnecessary medical delay. This, in turn, requires efforts on the part of hospital staff.

This paper reports the findings from a 3-year study on CPPs in Norway, where we investigate healthcare workers’ and patients’ experiences with CPPs. The aim of this paper is to describe how hospital staff cope with the increased focus on target times and deadlines and what measures and strategies hospitals and their staff have implemented to comply with the CPP target times. We also discuss the implications of the identified strategies for hospitals and for care delivery.

### Target times in cancer patient pathways

All Scandinavian countries have introduced CPPs. Denmark was the first country to do so, in 2007–2009. Norway introduced CPPs in 2015, based on the Danish model, and Sweden followed later in 2015. The UK has also introduced a similar solution through the ‘rapid diagnosis and assessment pathways’ for some cancer types, which are defined as timed diagnostic pathways (see, e.g. [15]). A common feature of these pathways across all these countries is that the steps are time-bound and that it is pertinent to comply with the set times.

The literature shows that the setting of times in cancer diagnostics and treatment, including in the CPPs, is driven by several reasons. One of the main reasons is to increase the survival rate by speeding up the diagnostic process, as early diagnostics has shown to improve the survival of cancer patients [16]. This was the main reason for introducing CPPs in Denmark [17, 18]. However, this is a less prominent reason in Norway, as Norway has a high survival rate [19]. Another reason relates to patients’ experiences with cancer diagnostics, in general, and their mental well-being, in particular. Minimising the time that patients spend waiting in uncertainty for test results and diagnosis would be beneficial for patients. This was a major reason for introducing CPPs in Norway, and it was also one of the main reasons in Denmark [20]. A third reason for establishing target times is to avoid differences in waiting times between patients. In Sweden, regional differences led to great variations in how long patients had to wait before they saw a physician. Therefore, one of the aims of introducing CPPs was to create more equal access to healthcare services. This reason was particularly prominent in Sweden [21, 22]; additionally, the NHS in the UK justifies the introduction of the cancer pathways with the goal of reducing variation in patient access to diagnostic and treatment options [15]. In Norway, too, the aim of reducing geographical variation was a motivation behind introducing CPPs.

### Efforts for meeting the target times

Complying with the target times set in CPPs is important for several reasons. However, the efforts made towards complying with the times must be understood within the context of hospitals facing great demand while grappling with a lack of resources. This, in turn, create bottlenecks in the organisation, delays in the patient pathway and, most probably, difficulties in meeting the target times. A Norwegian study investigated the reasons for prolonged diagnostic workup for stage I-II lung cancer [23]. The study found that the most important factors that delayed the diagnostic process was late referral for PET-CT and exercise tests, repetition of diagnostic procedures because of bronchoscopy failure and further investigations after PET-CT. Further, the findings showed that only 40% of the patients started treatment within the target time in the CPP. By investigating information in curative patients’ medical records, the authors discuss how the diagnostic pathway may be optimised. They conclude that changing the sequence of investigations could significantly reduce the time before start of treatment.

With regard to studies that have explicitly investigated hospital staff’s experiences with trying to meet the target times, we have only identified one study that touches upon the subject. The identified study investigated the implementation of CPPs in Sweden [21]. The authors showed that even though strong efforts were made to achieve the target times in one part of the system (e.g. the hospital), the lack of resources, competence or initiative in another part of the system hindered the processes and created bottlenecks in the system. This strongly influenced the extent to which the actors were motivated or able to act in accordance with the intentions of the CPPs, including meeting the target times.

The CPP target times are considered as national quality indicators, and meeting the target times may consequently be considered as an indicator of high-quality healthcare. However, measurements affect actors’ behaviour, and research has shown that performance measurements in healthcare can lead actors to game the system [24–26]. Gaming can be undertaken with the aim of gaining rewards or avoiding critique and negative publicity. Several studies have investigated how financial incentive schemes introduced to boost performance in the organisation may be gamed, for example, through upcoding [27] or other strategic behaviour [26]. However, financial incentive schemes could also be met with resentment [28]. To our knowledge, few studies have investigated instances of gaming to avoid negative publicity or sanctions. However, one study found that the levels of cleanliness in a hospital appeared to rise in the months leading up to an inspection, and a drop in the cleanliness levels were observed after the inspection period [26]. In order for such apparently strategic behaviour to take place, the staff obviously needs to be aware of an upcoming inspection. Staff working with CPPs are always indirectly subject to monitoring and inspection through the target times, and they are aware of this. This might mean that the staff exhibit strategic behaviour.

To summarise, two major trends work against each other in the pursuit of meeting the target times in CPPs: On the one hand, increasing demand and lack of resources create bottlenecks in the pathways, and on the other hand, professional and strategic ways are used to ensure compliance with the target times.

## Methods

### The study

The material, on which this article is based, is collected in a study on implementation of and experiences with CPPs among hospital staff, general practitioners and patients in Norway (2017–2020). The study has a qualitative cross-sectional design, and the data are collected through interviews. This article is based on interviews with hospital staff only. Four CPPs—for breast, prostate, lung and malign melanoma—are included in the study.

### Setting

The specialist healthcare sector in Norway (hospitals) is predominantly public, and is organised into four regional health trusts. Private providers may operate based on a contract with public hospitals, or in the market, where citizens themselves or insurance companies cover all costs. Cancer diagnostics and treatment are essentially handled by the public hospitals, but private providers offering services, such as medical imaging services, are used to overcome the lack of capacity in public hospitals.

CPPs are introduced in public hospitals without the formal involvement of private providers, even though the private providers may be the real starting point for some patients’ pathways. CPPs were introduced from January 2015, and the initial CPPs were created for the four ‘big’ diagnoses: breast, prostate, lung and colorectal cancer. Currently, there are 28 CPPs, including a CPP for unspecified serious symptoms which are indicative of cancer. When the health authorities instructed the hospitals to implement the CPPs, the hospitals were not provided with any extra resources. This means that they were required to work ‘smarter’ rather than only scale up their efforts.

### Data collection

For our study, we included five hospitals. We aimed at covering hospitals of different sizes and from different health regions. Two large, one medium and two small hospitals were included. All hospitals had implemented all 28 CPPs at the time of data collection. However, our study was limited to four of the CPPs. Table 1 depicts patient volume in each hospital for the included CPPs, and the percentage of the CPPs that met the target times. We present average figures for the period 2018–2019 because the data were collected over these years.

**Table 1:** Volume of patients included in the study’s four CPPs and the percentage that complied with the target time

Hospital	Number of patients, and percentage of CPPs complied with target times in parentheses^**b**^			
	Breast	Prostate	Lung	Malignant melanoma
Hospitals 1–2^a^	57 (80%)	86 (36%)	59 (41%)	75 (89%)
Hospital 3	275 (60%)	272 (49%)	151 (48%)	153 (92%)
Hospital 4	181 (63%)	111 (45%)	127 (71%)	95 (84%)
Hospital 5	440 (56%)	634 (43%)	172 (55%)	370 (72%)

^a^Statistics are combined for these two hospitals, because they are organised in the same Health Trust

^b^Average 2018–2019

Data were collected through semi-structured interviews, which are suitable for gaining insight into the lived worlds and experiences of actors [29]. We recruited informants through contact persons in each of the hospitals. The informants received written and oral information about the study prior to the interview and signed a consent form. The informants comprised physicians, nurses, pathway coordinators and low-level managers (mostly ward mangers) working with the four CPPs that were included in the study. In total, 60 hospital staff were interviewed. Most of the coordinators interviewed had a nursing background, while most of the managers were trained as physicians. Except for three interviews, the remaining were individual interviews. In the case of the three exceptions, two healthcare workers were interviewed together. Table 2 provides an overview of the informants, their role, and the distribution over the hospitals.

**Table 2:** Number of hospitals, informants, and their role

Hospital number	Role				Total
	Physician	Coordinator	Nurse	Manager	
Hospital 1	4	1		2	7
Hospital 2	3		2		5
Hospital 3	2	3	5	5	15
Hospital 4	5	5	3	1	14
Hospital 5	3	6		10	19

*N* = 60

We developed an interview guide that was used in all interviews with hospital staff. Table 3 shows the overall themes, as well as the subthemes that guided the interviews.

**Table 3:** Interview guide with overall themes and subthemes

Overall themes	Subthemes
Background information	• Position • Work experience • Involvement in work with CPPs
Comprehension of CPPs	• The practical meaning of CPPs to the informant • Opinion on the authorities’ objectives with CPPs
Implementation and training	• Information about CPPs • Experiences with the implementation • Training • Management’s role • Specific challenges in the implementation process
Organisational changes	• Similarities and differences in work practices before and after CPPs were introduced • Benefits and disadvantages with new work practices • The role of pathway coordinators • Organisation of multidisciplinary team meetings
Collaboration and communication	• Changes in collaboration/teamwork after the introduction of CPPs • Consequences for multidisciplinary work
Consequences for patients	• CPPs’ consequences for patients • Consequences for patients who are *not* included in a CPP
Reaching the objectives of introducing CPPs	• Is the goal that 70% of CPPs should comply with the target time met? • The relation between quality of care and meeting the target times
Further development	• Changes needed to improve how CPPs function, if any

Both authors conducted the interviews, and additional members from the project team participated in the interviews. The interviews lasted between 20 min and 1.5 h and were conducted between May 2018 and May 2019. All the interviews were tape-recorded and transcribed verbatim.

### Analysis

It became clear early on in the interviews that hospital staff were committed to making arrangements for rapid diagnostics, and that there was a strong focus on complying with the times stipulated in the pathways. Therefore, in the analysis, we wanted to explore in detail the informants’ experiences with the times. We used an analytical approach, described as a bricolage approach, which allowed us to draw on different analytic techniques. Such an approach implies a free interplay of techniques during the analysis, and one can combine approaches (e.g., narrative techniques, a counting statement indicating specific attitudes to a phenomenon, and working out metaphors) to capture key understandings [29]. In our case, the analysis started with both authors reading through all the transcripts to gain an overview of the material. The first author subsequently coded the interviews based on statements around time, strategies for meeting the target times and consequences of the focus on time. Microsoft Excel was used for organising the codes. The codes were initially synthesised under five main categories by the first author as a point of departure for discussion with the second author. When scrutinising the categories together, we agreed that one of the categories did not capture ‘a strategy’ and consequently it was omitted. The sub-categories within each of the main categories were also discussed. Some of them were merged, others were removed. During the analysis, our focus was to identify actions that in various ways represented ways of keeping the target times.

### Ethics

The study was approved by the Data Protection Official for Research in Norway (NSD) (Project no: 58724) and by the ethics committees of the health trusts included in the study. All participants were fully informed and provided their written consent for participation in the interviews.

## Results

We start this section by briefly describing the staff’s opinions and experiences with the CPP target times. Thereafter, we present the four main categories (Table 4), and explain them in detail by providing examples of ways of acting or strategies used to comply with the times, as described by the informants. Some of the ways and strategies were planned and formal, while others were created in an ad hoc manner and became routinised later.

**Table 4:** The four identified strategies for meeting the target times

Attention to meeting the target times		
**Strategies**	**New roles, more staff**	• Employ cancer pathway coordinators • Hire extra staff
	**Reorganising the workflow**	• Assess referrals continuously, on a day-to-day basis • Assign fixed time slots for examinations (e.g. MRI and lab work) • Reallocate hospital’s internal resources • Minimise the use of scarce resources
	**Gaming the system**	• Order several tests at the same time, without waiting for the test results • Use the phrase ‘CPP’ strategically to be prioritised in queues • Minimise time spent in one phase so that more time can be spent in another, or vice versa
	**Outsourcing services**	• Collaborate with other hospitals • Outsource to private providers

### Attention to compliance with the target times

The interviews showed that all the staff were highly aware of the target times in the CPPs. However, we found differences in compliance among the hospitals included in our study. The small hospitals had seldom experienced long waiting lists and problems with delayed diagnostic processes, while this was a rather common occurrence in the large hospitals. This may explain why informants from the small hospitals talked less about meeting the target times and were less concerned about it, even though they were aware of it. In contrast, many informants from the large hospitals expressed some form of cognitive stress. Some of them explained that they experienced constant pressure with regard to meeting the target times, and that they worked hard to achieve them:

I have the pressure on me all the time. I must make sure that all examinations are finished in due time. The ward is full, it is chaos (…) I am sending e-mails to the head of the department complaining, saying I cannot make it! (Cancer pathway coordinator, H3)

The findings indicate that particularly in the first phase following the introduction of the CPPs, much attention was given to meeting the target times. It also seems that managers were more preoccupied with ensuring that target times were met than were frontline workers. After a while, however, it received lesser attention, as explained by one manager:

In the beginning, there was much focus on the target times. We discussed it loudly, there was always a chat going on about the topic. We made adjustments, and the times decreased the first year. (…) but now, the times have increased, and we don’t manage to keep them, even if we want to. (Manager/physician, H3)

We found that the focus on meeting the deadlines was driven by two main reasons. First and foremost, it is important for the individual patient’s mental and physical health. In this regard, one coordinator (H5) stated, ‘we do it for the sake of the patient, not for ourselves’. Additionally, several of the informants emphasised that healthcare workers are highly aware that when a patient is diagnosed with cancer, he/she wants to receive treatment as fast as possible. The second reason is that the hospitals are assessed according to whether they comply with the target times. When such assessments are conducted and their findings are published publicly, some individuals become ‘extremely engaged in it’, as explained by a department manager (H5). Consequently, when you do not meet the target times, it leads to ‘a great commotion’.

One health trust introduced a computerised system for monitoring CPP times. This was considered helpful from an administrative point of view because it made it easier to gain an overview of all the CPP times and enabled them to follow up departments that were behind the target times.

### Introducing new roles and more staff

The interviews revealed that different actions and strategies were implemented to meet the target times. One overall strategy for dealing with the increased pressure on staff is to employ more people. Another strategy is to have a dedicated person to organise and coordinate the CPPs—a cancer pathway coordinator—and, thereby, indirectly relieve other staff of their tasks. In fact, a cancer pathway coordinator was appointed in all the hospitals in the study, while fewer hospitals had employed extra staff.

#### Employing cancer pathway coordinators

The pathway coordinator holds a central position in the CPP, as described by the health authorities. However, organising the position and formulating the job description falls under the purview of the hospital administration. We found that the coordinators conduct a variety of tasks and play a major role in facilitating compliance with the target times. The informants explained that the coordinator’s job is to organise and coordinate patients’ appointments, provide information to patients, call patients to inform them about their appointments, follow up when required (for example, if the test results are too late to meet the deadline), ensure that patients are given an appointment within the prescribed time frame, and answer all the questions of patients on the telephone. Coordinators also complete the electronic patient record (EPR) coding, thus documenting the completion of the various CPP phases. In our interview material, we found great diversity among coordinators and their position in the hospitals. A few were employed full time, others were employed for 50% of the time, and others simply had to incorporate the new tasks into their original job. The coordinators explained that they were required to pay close attention to the patients’ progress in the CPPs, and that they had the freedom to be ‘creative’, ‘pushy’ and ‘on the ball’ to ensure that the CPP was going according to plan. One coordinator explained that MRI examinations tended to introduce bottlenecks:

I am on the side-line, paying attention to patients, having them [investigations] quickly enough. So that the referrals do not lie there for 2-3 months. I know there is a pressure on MRI, and it is difficult to get an appointment. So, when I have a patient who needs an MRI, I am on the phone and fix it. (Pathway coordinator, H4)

Nurses who had the responsibility of coordinating activities prior to the introduction of CPPs noticed that the amount of work had increased. They talked about more coordination and more responsibility, as well as more ‘invisible work’, which refers to work that other people barely noticed but was necessary for the progress of CPPs.

#### Hiring extra staff

Hiring extra staff permanently or for a short period was another strategy identified for increasing the hospital’s capacity, and thereby, achieving the target times. When the hospitals were instructed to implement CPPs, no extra resources were provided to assist with the implementation. Therefore, most of the hospital departments that participated in our study did not have the financial means to employ new staff. However, in the departments that constituted the worst bottlenecks, such as the radiology department, more clinicians were hired. A radiologist from a small hospital explained that after the CPPs were implemented, they had to speed up the tempo of interpreting MRIs and CT scans to meet the CPP time frames. This was a challenge because there was already a shortage of radiologists. This particular hospital managed to employ an additional radiologist, but not all hospitals were successful in this respect. Some hospitals, especially those located in rural areas, had failed to recruit radiologists. They, therefore, had to develop other strategies for bypassing the bottleneck created by the lack of radiologists.

In one of the large hospitals, the capacity in the medical imaging department had been low for a long time. Their strategy was to hire additional radiologists for a short term to tackle the backlog, as explained by an oncology nurse:

The Directorate of Health writes reports about it [CPP times], and we were way down [on the list]. Maybe the worst in the country when it comes to describing the images, because there have been few resources in the mammography unit. They implemented some extraordinary measures, in the form of hiring radiologists. (Oncology nurse, H4)

### Reorganising the workflow

Another widely used measure for compliance with the target times was to reorganise the internal workflow in the hospitals. All cancer diagnostics involves a variety of examinations, and hence, the involvement of a great number of professions and departments. Typically, a diagnostic process consists of clinical examinations, medical imaging (e.g. MRI, CT and PET) and surgical biopsies conducted by pathologists. Some CPPs are relatively straightforward, while others can be complicated and require a great number of investigations. Consequently, there are many points of information, as well as patient handovers and potential bottlenecks. This necessitates efficient coordination and collaboration between actors, as well as a smooth workflow for ensuring the progress of the CPP. When CPPs were implemented, many of the departments in our hospitals found it necessary to reorganise the workflow to increase the speed of cancer diagnostics. Some of these changes had already been initiated, but in light of the CPPs, they were accentuated, along with a strong focus on time.

#### Assessing referrals continuously, on a day-to-day basis

One of the strategies for reorganisation is assessment of referrals on a day-to-day basis. Previously, many physicians had employed this strategy when it suited their work schedule. In order to meet the CPP target times, they were encouraged by the management to conduct these assessments daily. In this regard, a manager working with prostate cancer patients made the following statement:

Well, related to achieving the target time ‘start diagnostics’, we have tried to get all physicians who are involved in prostate cancer to assess referrals daily, and not wait until the end of the week. So, we can save some days. (Physician, H5)

In another hospital (H3), the Department of Lung Medicine had also initiated the same procedure. When a referral is received, either from general practitioners or another hospital department, and is marked as a CPP, it should be assessed within one day.

#### Assigning fixed time slots for examinations and surgery

Another measure to keep up the pace of the CPPs was to have time slots assigned specifically for patients in CPPs. Prior to the introduction of CPPs, many departments simply waited until a time slot was available for the required service. After the CPPs were introduced, such a strategy—or a lack thereof—was not feasible. The departments responsible for the cancer patients, therefore, conducted negotiations so they could have fixed times, e.g. a whole day with the radiology department for examination of breast cancer patients. An oncology nurse working with breast cancer patients elaborated:

It has always been difficult to get appointments for MRI. Now we have a fixed time. In particular for patients having adjuvant treatment – chemo – before surgery; they need to be examined with MRI, and now we have a fixed time slot for them, every Friday. That is very good, because we get the MRI much faster and they can start treatment earlier. (Oncology nurse 3, H3)

The study showed that some departments in the large hospitals had worked towards such an organisation before the CPPs were introduced, but that the CPPs and the related time monitoring triggered the need for more fixed examination times. For other hospitals, having fixed times was a new way of organising cancer diagnostics.

#### Reallocating hospital internal resources

A third strategy for reorganising the workflow was to reallocate resources on an ad hoc basis rather than on a fixed schedule (as with the previous strategy). Especially during holidays, it was difficult to meet the CPP target times. With regard to shortening the waiting times and ensuring compliance with the CPP times, informants explained that they were working hard to find solutions. An oncology nurse said:

This summer, it was put down an enormous effort. At one point of time, we had four weeks waiting time for surgery. We knew that we were beyond the deadline. We worked very hard trying to get operating rooms in other places, so we could reduce waiting times. (Oncology nurse 2, H3)

#### Minimising the use of scarce resources

A final example of changes in the workflow was to organise work so that staff who were in high demand would take part only in meetings and discussions where they were strictly necessary. This is an example of a strategy which can decrease delays. As explained earlier, radiologists were one such group of personnel who were in high demand, and pathologists were another such group of personnel who were in short supply. Pathologists play a pivotal role in examining biopsy specimens, and influence both the time when a diagnosis is established and the choice of treatment. One of the large hospitals had long struggled with a lack of capacity in the pathology department. When CPPs and target times were introduced, the procedures were changed for a certain type of biopsy, as explained by a physician working with lung cancer:

The hospitals are trying to make things better. We have for example removed the bone biopsies from the pathologists' schedule. The bone biopsies can be interpreted without a pathologist. We also try to label those others [biopsies] that can be conducted without a pathologist, so that we rather use the pathology-days for other patients. (Physician, H5)

### Gaming the system

The two categories described above are formal, thought-through and known strategies used for increasing hospital capacity and efficiency. The third category of strategies is distinct from the previous two because they are closely related to the CPPs and are not usually a part of formal procedures and plans.

#### Order several tests at the same time, without waiting for test results

One of the strategies was to order several tests and examinations at the same time, without waiting for the results. Previously, physicians would normally wait until they received the test results, and based on the findings, decide if it was necessary to order more tests and examinations. However, with the tight time schedule of CPPs, many informants found that it was impossible to wait for the results if you wanted to meet the deadlines. They would, therefore, order several tests at the same time. One of the hospital’s radiology departments raised the concern that patients may go through unnecessary clinical examinations, and risk being exposed to unnecessary radiation, because of the time pressure. This is neither beneficial for the patient’s health, nor beneficial for optimal resource utilisation in the hospital. However, we did not find too many examples of such practices. In general, informants stressed that they stayed true to the diagnostic guidelines and would comply with them, even if it meant violating the target times.

#### Using the phrase ‘CPP’ for priority in queues

Another much more frequently used strategy which many of the informants talked about was using the phrase ‘cancer patient pathway’ as a crowbar to get rid of bottlenecks and be prioritised in queues. Queues would typically form for investigations where there were shortage of equipment or personnel with the required competence. Investigations and surgeries for patients with non-fatal diseases would be postponed so the diagnostic process for cancer patients could proceed rapidly. Examples of patients who were downgraded were breast cancer patients waiting for reconstructive surgery, and patients in need of minor plastic surgery, to mention a few. There were many examples of situations where staff working with CPPs would emphasise that the patient was in a CPP and, therefore, needed to be prioritised in a queue. A physician explained that resources became more available to their patients after the introduction of CPPs:

If we have any bottlenecks, where things come to a stop in the pathway, we can insist on having the necessary resources because CPPs are mandatory measures by the authorities (…) When it is a CPP, doors are opened and priorities are in your favour when you have the CPP. (Physician, H3)

A pathway coordinator (H2) reported the same experience: When she called to book an examination and explained that the patient was in a CPP, the receiver understood that this patient needed to be prioritised. However, although the CPP patients were often prioritised, sometimes, there were queues of only CPP patients that made prioritization more difficult.

### Outsourcing services

The fourth identified category of strategies was to outsource activities and services to either another (public) hospital or a private provider.

#### Collaborating with/seeking assistance from another hospital

There were some examples of strategies that involved outsourcing activities to other hospitals that specifically related to meeting the target times. In one example from a small hospital in our study, it had closed some of its services during the summer holiday and, therefore, needed to collaborate with the neighbouring hospital for particular surgical procedures and specific tests which were part of the diagnostic workup. The informants explained that if the patient had to wait until after the holiday, they would not meet the target times. However, the informant also stressed that it would not be desirable for the patient to wait for such a long time. Another example was from a large hospital, which also had to acquire the services of another hospital to meet the target times. One melanoma pathway coordinator explained that they had to seek the assistance of two other hospitals in the Health Trust:

I think we have the sentinel node biopsies* on Tuesdays and Wednesdays [in our own hospital], and now we had to get an extra examination day in Hospital 6, because we do not manage to handle the queue within the deadlines. And in addition, we have surgery in hospital 7 too, to meet the target times.* (Pathway coordinator, H5)

In another hospital (H4), a coordinator explained that she received referrals for cancer diagnostics for melanoma. She would review them before handing them over to the physicians, who decide if the patient is eligible for the CPP. If there was a long waiting time at the hospital, she would write on the referral note: ‘can this patient be examined in hospital 17? Or hospital 15? Or Hospital 16?’ In this way, she tried to help to achieve the target times, by suggesting different options for examination.

#### Outsourcing to private providers

In addition to receiving assistance from other hospitals, there were also examples of hospitals that use private providers to bypass bottlenecks and to free up capacity. Particularly with regard to radiology services, the use of private providers was common. One radiologist said:

We use private providers, and we have been allowed to increase the amount of work they do for us (…) With regard to the collaboration with private providers…everyone, or many, say that it's no good, but I think it is very good. (Physician H5)

The resistance against using private providers most probably comes from a strong tradition in Norway according to which healthcare is a public responsibility and private providers traditionally have a minor role. However, to overcome the lack of capacity, many hospitals turn to private providers. The interviews indicate that it is the patients who are least likely to have cancer that are transferred to private providers. The difficult and advanced cases remain in the public hospitals for diagnostics.

## Discussion

Norwegian hospitals did not receive additional funding or detailed instructions for the implementation of CPPs. They, consequently, had to invent their own measures and strategies to ensure the CPPs’ progress. In the previous section, we have presented the variety of strategies applied for meeting the CPP target times. By outlining the diversity of these strategies, we have aimed to show that organisational reforms, such as the introduction of CPPs, are not possible without the hard work of frontline staff and may have unintended consequences.

Studies on the implementation of clinical pathways agree that both reorganisation of current work processes on an organisation level [21] and behavioural changes on an individual level [30] are required to achieve the desired outcome. Implementation may also be expensive and, therefore, raises the question of financing the new activities [13]. The possibility of and enthusiasm towards undertaking the necessary changes are furthermore affected by the context of implementation, such as the lack of outcome expectancy, time constraints and insufficient staffing [31, 32]. It is, therefore, challenging to fully understand the implementation of CPPs. In our study, we have focused on one specific element of the CPP—compliance with target times. We found several examples of how organisational changes and changes in staffing have been made to overcome the lack of personnel and resources, and how this can, in turn, jeopardise compliance with the target times. The first strategy, which was also prescribed by the authorities, was to assign a pathway coordinator. In our hospitals, all the personnel who became coordinators were already employed at the same hospital. They either had a similar position and were given more duties, or were relocated from a different unit and assigned new tasks and responsibilities. Furthermore, hospitals saw the need to hire more staff, for example, radiologists, who formed a group of personnel that most of the hospitals were short of. These strategies involving changes in staffing may be seen as a way of overcoming the barrier presented by the lack of resources.

The strategies within the category ‘reorganising the workflow’ are also clear examples of organisational changes. The rationale behind clinical pathways is, de facto, to depict a consistent workflow for care delivery [33]. However, in the case of CPPs in Norway, the hospitals were given a lot of freedom with regard to how they could reorganise their workflow, and this paved the way for creative and varied solutions. It is well known that allowing for flexibility in pathway organisation can increase the chances of successful implementation of CPPs [31, 34]. Reorganisation of workflows required organisational changes, but at the same time, there were also examples of individual healthcare workers who had to change their behaviour. A striking example is how physicians previously would typically assess referrals and report their decisions once a week. This strategy provided them with a high level of flexibility and autonomy. However, to meet the target times in the CPP, physicians were required to change pace and assess referrals several times a week. Thus, more detailed work instructions pose a challenge to physicians’ autonomy. It is likely that this development continues in healthcare, in general, given that more and more work processes have become streamlined.

CPPs and the efforts of hospitals and their staff to meet the target times has led to strains on diagnostics capacity. We found that our hospitals both collaborated with other (public) hospitals to increase capacity as well as outsourced services to private providers. The first approach was applied by all hospitals, regardless of size, while the latter seems to have been more prominent among the larger hospitals. Capacity pressure results in ‘crowding-out’ effects when CPP patients are prioritised to meet target times. The effects of CPPs on non-cancer patients has not been a focus of this article, but informants in the study explained that when cancer diagnostics is prioritised, other patient groups without diagnostic target times are given less priority. This is in line with other studies that focus on crowding-out effects as unintended, or at least unwanted, consequences of prioritising certain patient groups [22, 35, 36]. More extensive use of private providers may, however, be a remedy for low-priority patients, that is, patients with less serious, not life-threating conditions. Hospitals may refer them to private providers and use their scarce resources on the most complicated and advanced patient cases. In this way, the introduction of CPPs can contribute to a discussion on how to utilise resources and create a sound division of labour across all healthcare providers.

The last category of strategies comprises ways of acting to game the system. Previous research underscores that individuals, or collectives of individuals, may change behaviour when they are measured [24–28]. The CPP target times are national quality measures and figures for each hospital, and each CPP is published publicly monthly. It is, therefore, not surprising that hospital staff find creative ways to comply with the target times. Our data revealed, unsurprisingly, the following tendency: staff in the larger hospitals that experienced more capacity problems were more likely to exert strategic behaviour than were those in hospitals with better capacity. The literature on gaming the healthcare system predominantly deals with actors manipulating the system to present themselves and their organisation as better than they are in real life. However, in our case, healthcare workers instead act to prevent looking bad.

## Conclusion

CPPs are introduced to shorten waiting times, to eliminate unwanted variations and to improve patient safety and satisfaction. In this article, we have focused only on the shortening of waiting times, and the efforts and strategies used to meet the target times. Target times are an important element of CPPs, and hospital staff are highly aware of them. Meeting the target times is seen as important both for the sake of the patient and for the reputation of the hospital. Waiting times are decreasing for most diagnoses, and hospital staff relate this to the introduction of CPPs. We found that hospitals and their staff have developed different strategies for complying with the target times. These strategies include internal re-organisations within hospitals, individual behavioural changes and redistribution of services to providers outside the hospital. Our study shows that the introduction of CPPs have generated work for frontline staff, as well as managers, but they are not being compensated for it. Additionally, CPPs and the strong focus on target times may also have negative consequences for other patient groups. Therefore, when introducing future care pathways, it is necessary to consider both the costs and the potential gains, and to plan for the extra effort needed to implement organisational change.

## Data Availability

The dataset used during the current study are available from the corresponding author on reasonable request.

## Abbreviation

CPP: Cancer patient pathways
